# The Influence of Hydrogen-Charging Current Density and Temperature on Hydrogen Permeation and Hydrogen Embrittlement Susceptibility of 4130X Steel

**DOI:** 10.3390/ma18153448

**Published:** 2025-07-23

**Authors:** Caijun Xu, Fang Wang, Jiaqing Li

**Affiliations:** 1 Fujian Boiler and Pressure Vessel Inspection and Research Institute, Fuzhou 350008, China; fjtjxucaijun@163.com; 2 College of Chemical Engineering, Fuzhou University, Fuzhou 350116, China; jiaqing@fzu.edu.cn

**Keywords:** 4130X steel, hydrogen permeation, hydrogen-induced degradation, temperature threshold

## Abstract

Chromium-molybdenum steels are extensively used in manufacturing large-volume seamless hydrogen storage vessels, but they still suffer from the hydrogen embrittlement problem. In this study, electrochemical cathodic hydrogen charging is utilized to investigate the hydrogen embrittlement of 4130X steels, with emphasis on the influence of charging current density and temperature on hydrogen permeation and hydrogen embrittlement susceptibility. The hydrogen penetration rate and hydrogen diffusion coefficient of 4130X steel both increase with an increase in hydrogen-charging current density and temperature. The results demonstrate that the degree of hydrogen-induced degradation in tensile ductility is more marked with increasing hydrogen-charging current density, while the hydrogen embrittlement index exhibits a peak at a temperature of 308 K, in which brittle patterns like quasi-cleavage surfaces and crack formations occur. These findings are crucial for understanding hydrogen-induced embrittlement and determining test temperatures of hydrogen-related engineering material applications.

## 1. Introduction

In alignment with the country’s commitment to achieving the “Dual Carbon” goal, hydrogen has gradually emerged as a viable solution, with the potential to revolutionize the global energy economic system [1,2]. As an exceptionally versatile fuel, hydrogen offers the significant advantage of net-zero end-use emissions [3], positioning it as an ideal candidate for driving decarbonization across various high-emission sectors, including transportation, industry, electricity generation, and heat production. However, during hydrogen storage and transportation, metals and alloys usually face the problem of hydrogen embrittlement (HE) [4,5,6], that is, the invasion of hydrogen atoms degrades the strength, ductility, and fracture toughness of the material. In the early 19th century, researchers first discovered the HE phenomenon and theorized that “hydrogen obstructs the movement of iron molecules” [7]. Over the past decades, thousands of articles on the HE topic have been reported [8], leading to the proposal, rigorous debate, and validation or refutation of numerous HE theories.

HE can be divided into two categories: reversible and irreversible [9]. Reversible hydrogen embrittlement means that when all the hydrogen in a material is removed, the brittleness will disappear. Irreversible HE refers to the fact that the phenomena related to HE cannot be eliminated, including bubbles, aggregates, and cracks in the heat-affected zone of welding [10]. There are several important theories including hydrogen-enhanced decohesion (HEDE) [11], adsorption-induced dislocation emission (AIDE) [12], and hydrogen-enhanced localized plasticity (HELP) [13], and so on. In the 1960s and 1970s, Troiano [14] and Oriani [15] invoked the concept of HEDE, presuming that hydrogen atoms can weaken the bond between atoms in steel, resulting in grain boundary (GB) cleavage or intergranular crack propagation. Based on the observation of fracture morphology characteristics, Beachem [11] proposed the HELP mechanism, which postulates that there is a direct relationship between hydrogen-induced cracking and the microstructure of materials. Subsequently, the theory was further developed by Birnbaum [16], Robertson [17], and Sofronis [18]. According to the developed model, a large number of hydrogen atoms can gather at the crack tip due to stress concentration, promoting dislocation activity and local plasticity nearby the crack tip, and eventually leading to crack initiation. In 1976, the AIDE theory was proposed by Lynch [12,19,20], which suggests that hydrogen adsorption in the metal subsurface can weaken the interatomic bonds over several atomic distances, and thereby promotes the simultaneous generation of a dislocation core alongside surface step structures. This dislocation emission induces strain concentration, causes the formation of microvoids in the front of the crack tip, and ultimately leads to crack propagation via microvoid coalescence.

Chromium-molybdenum (Cr-Mo) steels are extensively used in manufacturing large-volume seamless hydrogen storage vessels. However, as hydrogen storage pressures increase, Cr-Mo steels face significant challenges, including mechanical property degradation, reduced ductility and plasticity, and accelerated fatigue crack propagation caused by the aforementioned HE [21]. For example, Matsunaga et al. [22] investigated the performance of Cr-Mo steels under 115 MPa hydrogen and nitrogen environments at various temperatures using slow strain rate tensile (SSRT) experiments. Their findings revealed that tensile strength did not decrease with increasing temperature. In a hydrogen environment, the fracture surfaces exhibited prominent cracks, and fracture shrinkage was significantly lower compared to specimens tested in a nitrogen environment. Despite an extensive body of research on the HE behaviour of Cr-Mo steels [23,24], the previous literature mainly used gaseous hydrogen charging to simulate hydrogen-bearing environments. The gaseous hydrogen-charging method aligns with the actual service conditions of steels; however, the kinetic mechanisms governing the dissociation of hydrogen molecules into hydrogen atoms remain inadequately understood. Furthermore, the impact of the steel surface state on the generation of hydrogen atoms is not yet well clarified. These unresolved issues raise concerns about the reliability of using gaseous hydrogen charging to study hydrogen-related phenomena in Cr-Mo steels.

By comparison, electrochemical cathodic hydrogen charging has been globally recognized as a standard testing approach. This approach enables the acquisition of stable and reliable measurement results, such as electrochemical hydrogen-charging current density over time, under carefully controlled conditions. Therefore, electrochemical cathodic hydrogen charging is applied herein to explore the HE of Cr-Mo steels, emphasizing the impact of charging current density and temperature on hydrogen permeation behaviour and HE sensitivity. As a typical type of Cr-Mo steel, 4130X steel is selected as the experimental subject. The organization of this study is presented as follows: The details of experimental materials and methods are described in Section 2. Hydrogen permeation and hydrogen-induced degradation in the tensile properties influenced by charging current density and temperature are analysed and discussed in Section 3, followed by the main conclusions in Section 4.

## 2. Materials and Methods

### 2.1. Material Preparation

**Figure 1:**
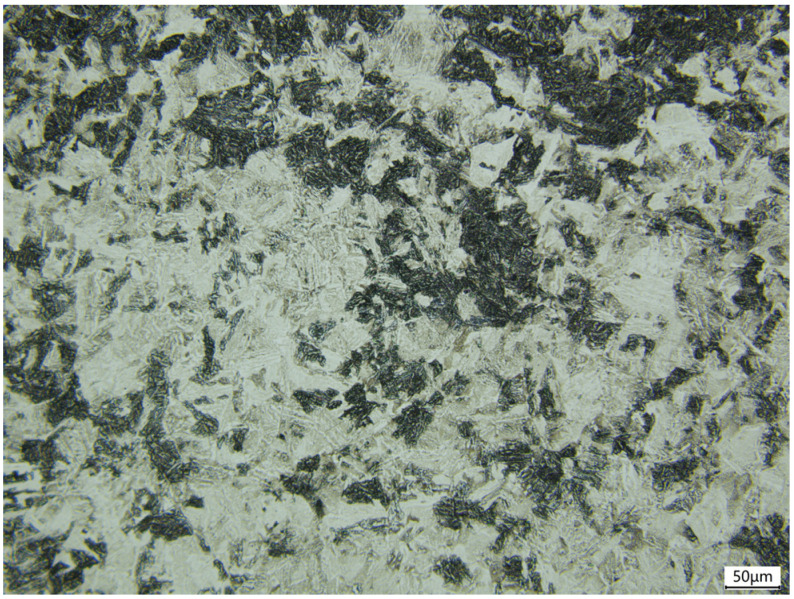
The microstructure of 4130X steel characterised by OM.

The chemical compositions of the 4130X steel employed in this research are listed in Table 1, and the steel’s microstructure, characterized by using a WUMO WMJ-9370 optical microscope (OM) (Hefei, China), is illustrated in Figure 1. As observed, the fundamental structure of 4130X steel consists of a massive ferrite matrix interlaced with pearlite and bainite phases.

### 2.2. Hydrogen Permeation Tests

**Figure 2:**
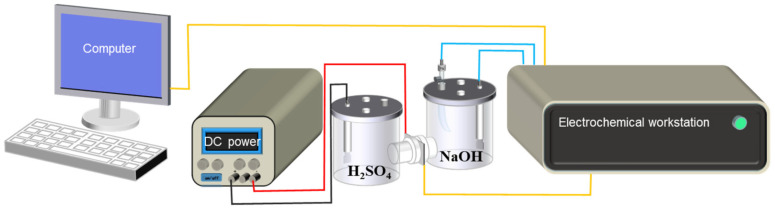
Schematic illustration of electrochemical hydrogen permeation cells [24].

Electrochemical hydrogen permeation experiments were conducted by using Devanathan–Stachurski cells [25,26,27], which typically include a hydrogen-charging cell containing 0.5 mol/L H_2_SO_4_ solution, a hydrogen oxidation cell with 0.1 mol/L NaOH solution, and a sample serving to separate the two cells as shown in Figure 2. Prior to hydrogen permeation, all 4130X steel samples were processed by wire cutting and ground into a shape of 30 mm × 30 mm × 1 mm. Subsequently, the hydrogen oxidation side of the sample was electroplated by nickel to reduce the background current. To investigate the effect of hydrogen-charging current density, an external DC power supply was used to adjust the current density of the electrochemical hydrogen-charging side from 10 mA/cm^2^ to 50 mA/cm^2^. Meanwhile, variation in oxidation current density at the hydrogen oxidation cell was monitored by an electrochemical workstation to obtain hydrogen permeation parameters. In order to elucidate temperature-dependent hydrogen permeation, experimental temperatures were set at 293 K, 298 K, 303 K, 308 K, and 313 K by controlling a water bath.

### 2.3. In Situ Tensile Tests

In situ hydrogen-charging SSRT tests were carried out by using a WANCE ETM204C tensile tester (Shenzhen, China) coupled with an electrochemical cell comprising an inner and an outer chamber [26]. The tensile samples with gauge dimensions of 30 mm × 6 mm × 2 mm were mounted on a tensile machine, and immersed in the inner chamber of the electrochemical cell with an electrolyte solution of 0.5 mol/L H_2_SO_4_ with 0.2 g L 1 CH_4_N_2_S for hydrogen charging. An external DC power supply was connected with the sample to adjust the current density of in situ hydrogen charging from 10 mA/cm^2^ to 50 mA/cm^2^. In the meantime, a constant-temperature liquid was used in the outer compartment of the electrochemical cell to regulate the experimental temperature between 293 K and 313 K at 5 K intervals. All SSRT tests were performed at a constant strain rate of 10^−6^ s^−1^, and were repeated three times for each experimental condition.

After tensile tests, several parameters, such as reduction in cross-sectional area Ψ, elongation to failure δ, and HE index $F_{H}$, were calculated and assessed by the following formulas:

(1) $$\Psi =\frac{S_{0}-S_{f}}{S_{0}}\times 100\%$$

(2) $$\;\delta =\frac{L_{f}-L_{0}}{L_{0}}\times 100\%$$

(3) $$F_{H}=\frac{\Psi_{air}-\Psi_{hydrogen}}{\Psi_{air}}\times 100\%$$

where $S_{0}$ and $S_{f}$ represent the initial cross-sectional area of the sample and the area of its minimum cross-section after fracture; $L_{0}$ and $L_{f}$ are the original and ultimate gauge length, respectively; and $\Psi_{air}$ and $\Psi_{hydrogen}$ are the reductions in cross-sectional area in the air and hydrogen-charging environment.

## 3. Results and Discussion

### 3.1. Hydrogen Permeation Behaviour of 4130X Steel

#### 3.1.1. The Effect of Hydrogen-Charging Current Density on Hydrogen Permeation

**Figure 3:**
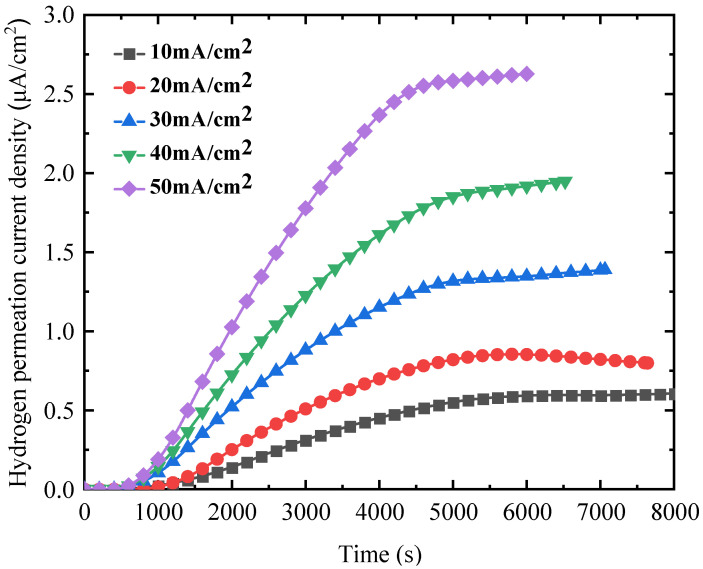
Hydrogen permeation curves versus time at different hydrogen-charging current densities.

Figure 3 shows the hydrogen permeation curves of 4130X steel under various hydrogen-charging current densities at ambient temperature (300 K). It is evident that the permeation current density equals zero at t=0 s. After a while it experiences a significant increase with time, and eventually reaches a quasi-steady-state value $i_{\infty }$. When the hydrogen-charging current density is high, the time required to reach the quasi-steady-state becomes short, and the quasi-steady-state value $i_{\infty }$ becomes large. According to the basic principle of electrochemical hydrogen penetration, hydrogen atoms are produced within the hydrogen-charging cell, diffuse into the hydrogen oxidation cell, and are all oxidized to generate permeation current. Therefore, the measured permeation current density versus time directly reflects the process of hydrogen diffusion. In order to quantify this process, several hydrogen permeation parameters such as the hydrogen diffusion coefficient $D_{eff}$ and hydrogen penetration rate $J_{H}L$ can be derived by using the constant concentration model and time lag method [28,29]:

(4) $$D_{eff}=\frac{L^{2}}{6t_{L}}$$

(5) $$J_{H}L=\frac{i_{\infty }L}{nF}$$

where L is the thickness of the sample; $t_{L}$ is the lag time, in which the corresponding permeation current $i_{t}$ = 0.63$i_{\infty }$; n is the charge number; and F is the Faraday constant with a value of 96,500 C/mol.

The hydrogen permeation parameters derived as a function of hydrogen-charging current density are listed in Table 2. With an increase in hydrogen-charging current density, the quasi-steady-state value $i_{\infty }$ increases prominently. Also, the hydrogen diffusion coefficient $D_{eff}$ increases with the charging current, which is consistent with a previous study [30].

#### 3.1.2. The Effect of Hydrogen-Charging Temperature on Hydrogen Permeation

**Figure 4:**
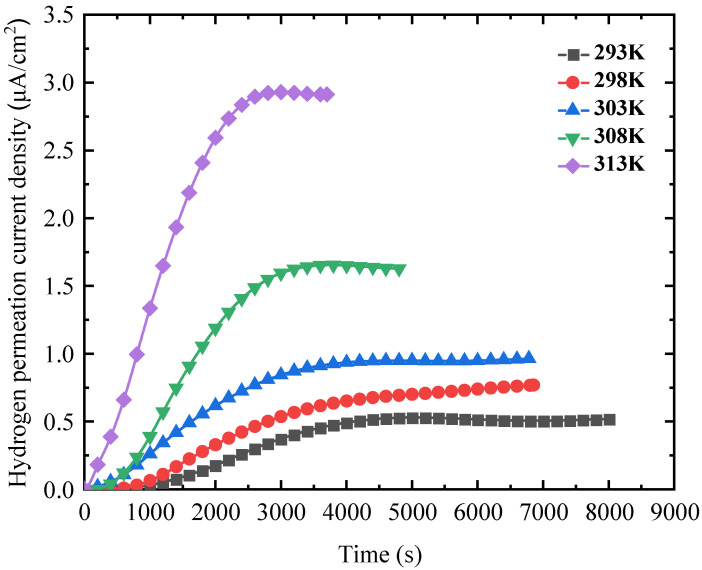
Hydrogen permeation curves versus time at various temperatures.

To explore the influence of temperature on the hydrogen permeation behaviour of 4130X steel, hydrogen permeation tests were performed at 293 K, 298 K, 303 K, 308 K, and 313 K, with a charging current density of 10 mA/cm^2^. The hydrogen permeation transient versus time as a function of temperature is depicted in Figure 4. As shown, increasing the temperature significantly enhances the quasi-steady-state value $i_{\infty }$ while also reducing the time required for hydrogen permeation to reach equilibrium. The hydrogen permeation parameters, including hydrogen diffusion coefficient $D_{eff}$ and hydrogen penetration rate $J_{H}L$, are again calculated and presented in Table 3. These parameters all exhibit an upward trend with increasing temperature.

**Figure 5:**
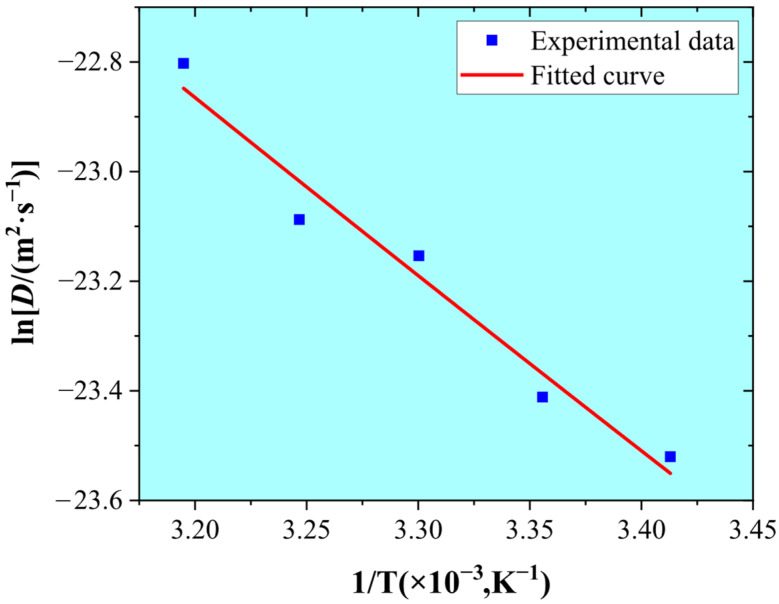
Hydrogen diffusivity as a function of the inverse of temperature.

By comparing the results in Table 2 and Table 3, it is evident that the increase in $D_{eff}$ caused by the charging current density seems insignificant, while a substantial increase in $D_{eff}$ is observed with temperature. Theoretically, diffusion coefficient $D_{eff}$ as a function of temperature T follows the Arrhenius equation [31,32,33]:

(6) $$D_{eff}=D_{0}\times \exp\left(\frac{-E_{D}}{RT}\right)$$

where $D_{0}$ is the pre-exponential factor; R is the gas constant; T denotes the temperature; and $E_{D}$ is the activation energy of hydrogen diffusion. By substituting the experimental data into the Arrhenius relationship, Figure 5 shows the obtained hydrogen diffusivity as a function of the inverse of temperature. The fitted values of $D_{0}$ and $E_{D}$ are 2.074 × 10^−6^ m^2^/s and 26.973 kJ/mol, respectively, which are consistent with the previous literature results [34,35,36].

### 3.2. Hydrogen-Induced Degradation in Mechanical Properties of 4130X Steel

#### 3.2.1. The Effect of Hydrogen-Charging Current Density on Mechanical Properties

**Figure 6:**
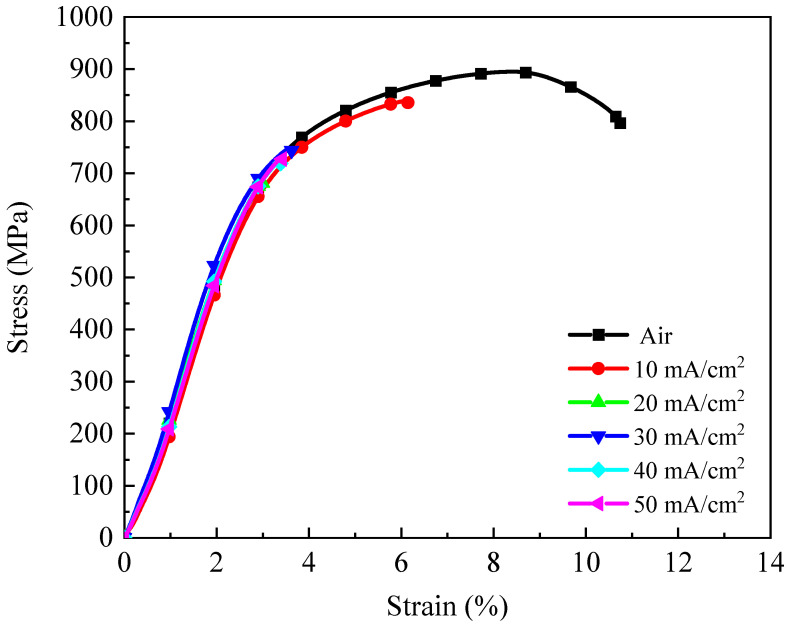
The stress–strain curves of 4130X steel at various hydrogen-charging current densities.

The stress–strain curves of 4130X steel at multiple hydrogen-charging current densities are depicted in Figure 6. It can be seen that the tensile strength and ductility of the hydrogen-charged samples are significantly lower than those of tensile samples in air. For instance, the tensile strength and elongation to failure of 4130X steel in air is 895.15 MPa and 10.74%, while these values are decreased to 839.51 MPa and 6.14%, respectively, under a hydrogen environment with a charging current density of 10 mA/cm^2^. The detrimental impact of hydrogen on the mechanical properties of Cr-Mo steel has been thoroughly documented in previous studies [21,22,37,38].

**Figure 7:**
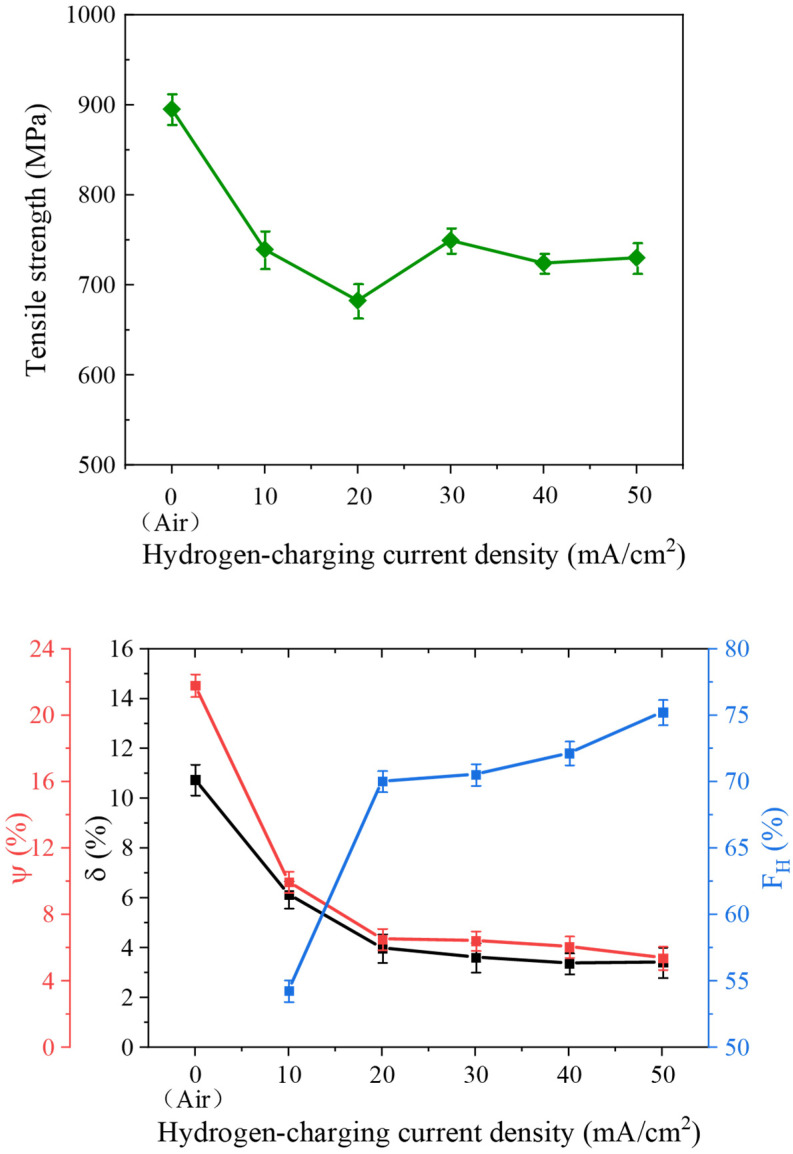
The dependence of mechanical property parameters on hydrogen-charging current density.

To quantitatively evaluate the impact of hydrogen-charging current density on the HE susceptibility of 4130X steel, several key parameters—including elongation at failure *δ*, cross-sectional area reduction *Ψ*, and HE index *F_H_*—were computed and are presented in Table 4 and Figure 7. It is widely acknowledged that as elongation at failure and cross-sectional area reduction decrease, a material’s susceptibility to HE increases. It is obvious from Table 4 that the δ and Ψ of each sample decrease with an increase in hydrogen-charging current density, indicating the aggravation of HE behaviour. Furthermore, the $F_{H}$ versus charging current density in Figure 7 again demonstrates that the hydrogen-charging current density facilitates the occurrence of HE, with the $F_{H}$ reaching its maximum at a charging current density of 50 mA/cm^2^.

#### 3.2.2. The Effect of Hydrogen-Charging Temperature on Mechanical Properties

**Figure 8:**
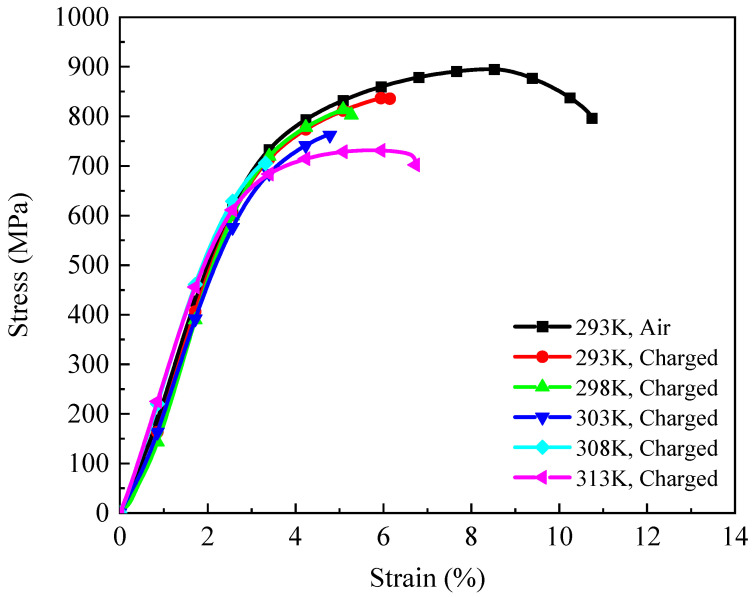
The stress–strain curves of 4130X steel at various hydrogen-charging temperatures.

Figure 8 illustrates the stress–strain curves of tensile samples under hydrogen charging at temperatures ranging from 293 K to 313 K. For comparison, the mechanical response of an uncharged 4130X sample is also included [39,40,41,42]. Notably, at 293 K, the tensile strength and ductility are reduced to 839.51 MPa and 6.14%, respectively, under a hydrogen environment. Additionally, for other hydrogen-charging temperatures, hydrogen-induced degradation in both tensile strength and ductility is apparent from the measured stress–strain curves.

**Figure 9:**
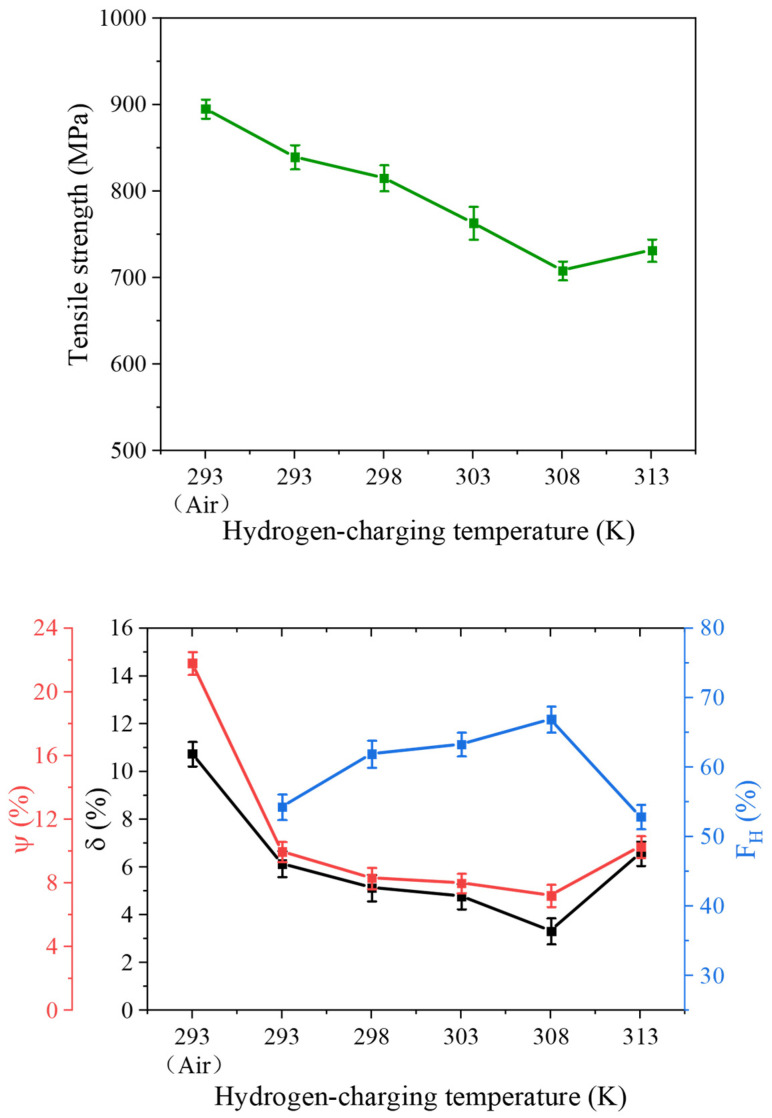
The dependence of mechanical property parameters on hydrogen-charging temperature.

Table 5 and Figure 9 present the variation of mechanical property parameters with hydrogen-charging temperature. As evident, the ductility degradation caused by hydrogen charging appears to be temperature-dependent. Specifically, the δ and Ψ values of hydrogen-charged specimens initially decrease from 6.14% and 9.97% to 3.33% and 7.22% as the temperature increases from 293 K to 308 K but exhibit an upward trend when the temperature rises from 308 K to 313 K. Both parameters reach their minimum at 308 K, indicating that hydrogen-induced degradation is most severe at this temperature. Higher HE indices generally signify more pronounced HE susceptibility. The HE index versus temperature plot in Figure 9 further confirms that 308 K serves as the critical temperature threshold *T_HE,max_*, where the HE susceptibility is the highest, i.e., 66.88%, and the embrittlement effect is less above and below this temperature. The observation of the temperature threshold occurring at 4130X steel can be compatible with the experimental data obtained from other structural materials in the previous literature [41,42,43,44].

### 3.3. Hydrogen-Induced Transition in Fracture Morphology of 4130X Steel

#### 3.3.1. The Effect of Hydrogen-Charging Current Density on Fracture Morphology

**Figure 10:**
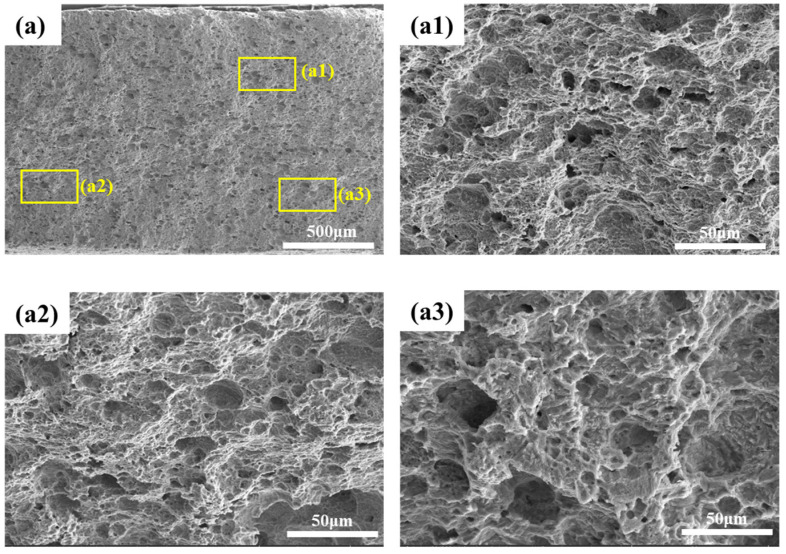
(**a**) Microscale SEM images of the fracture surfaces of non-charged specimens: (**a1**) High-resolution morphology of the scanning electron microscope image in the upper right region; (**a2**) High-resolution morphology of the scanning electron microscope image in the lower left region; (**a3**) High-resolution morphology of the scanning electron microscope image in the lower right region.

**Figure 11:**
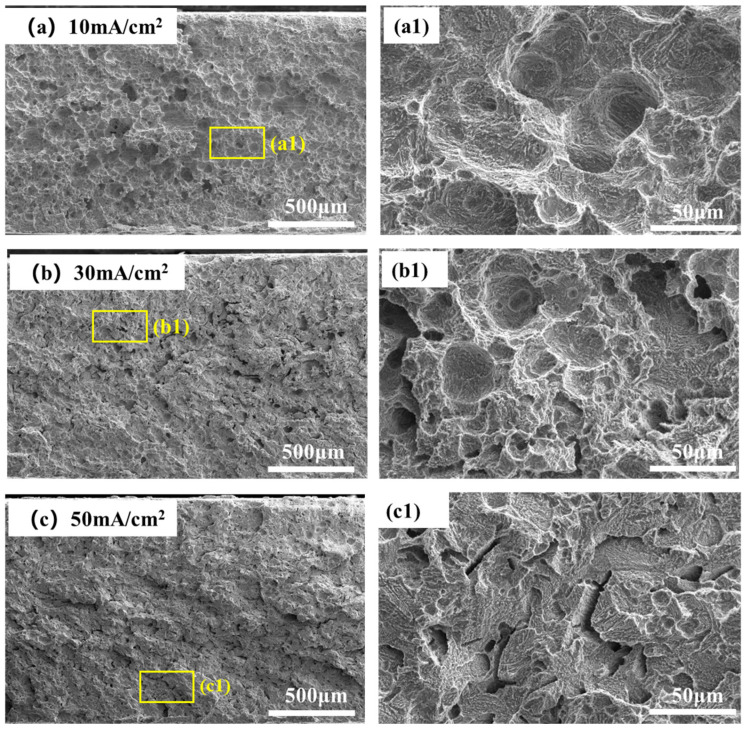
Microscale SEM images of the fracture surfaces of hydrogen-charged specimens at various hydrogen-charging current densities: (**a1**) corresponds to the high-magnification image of the yellow box in (**a**); (**b1**) corresponds to the high-magnification image of the yellow box in (**b**); (**c1**) corresponds to the high-magnification image of the yellow box in (**c**).

The fracture morphologies of 4130X steel after SSRT tests in air and at various charging current densities are shown in Figure 10 and Figure 11. As shown in Figure 10, the fracture surface microstructures of tensile samples are predominantly ductile, featuring numerous voids and dimples. In contrast, when a hydrogen-charging current density of 10 mA/cm^2^ is applied, the dimples and voids in the hydrogen-charged samples shown in Figure 11a become sparser and shallower. With a further increase in hydrogen-charging current density to 30 mA/cm^2^, in addition to dimples, some cracks are formed and observed at the fracture surfaces. At a hydrogen-charging current density of 50 mA/cm^2^, there are almost no dimples; instead, the fracture surfaces manifest with secondary cracks and quasi-cleavage planes. The emergence of a quasi-cleavage fracture is an evident support of the embrittling effect of hydrogen at a higher hydrogen-charging current, which is in accordance with mechanical property parameters in Figure 7. Previous studies have shown that hydrogen damage can occur when the hydrogen concentration exceeds the critical value [14,45]. As measured by hydrogen permeation tests in Figure 3, a high hydrogen-charging current density means higher hydrogen concentration, which leads to the transition in fracture mode from ductile fracture to quasi-cleavage fracture [46].

#### 3.3.2. The Effect of Hydrogen-Charging Temperature on Fracture Morphology

**Figure 12:**
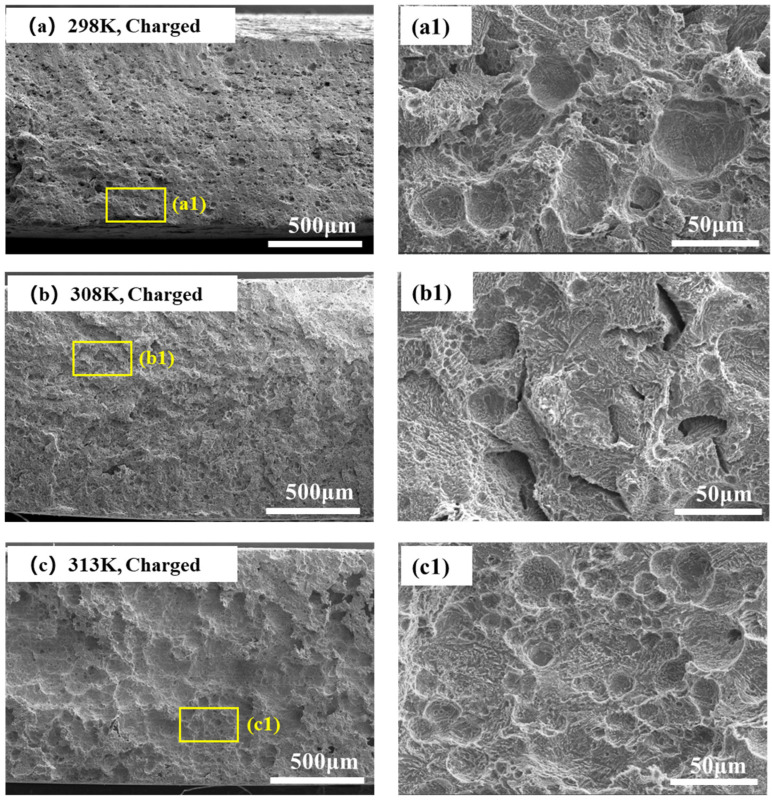
Microscale SEM images of the fracture surfaces of hydrogen-charged specimens at various hydrogen-charging temperatures: (**a1**) corresponds to the high-magnification image of the yellow box in (**a**); (**b1**) corre-sponds to the high-magnification image of the yellow box in (**b**); (**c1**) corresponds to the high-magnification image of the yellow box in (**c**).

Figure 12 shows the fracture morphologies of hydrogen-charged samples at different hydrogen-charging temperatures. The fracture morphology is dominated by dimples at the hydrogen-charging temperatures of 293 K and 298 K. With a further increase in temperature to 308 K, the ductile fracture characteristics almost disappear, and the quasi-cleavage characteristics appear at the fracture surface accompanied by the occurrence of secondary cracks. When the temperature rises to 313 K, the fracture morphology again exhibits a ductile pattern as evidenced by numerous dimples and voids, as shown in Figure 12c.

Based on the aforementioned fractographic analysis, it is inferred that ductility decreases as the temperature rises to 308 K, after which it increases with further temperature elevation. At 308 K, quasi-cleavage planes and cracks are observed, indicating that HE susceptibility reaches its peak, which aligns with the HE index depicted in Figure 9.

**Figure 13:**
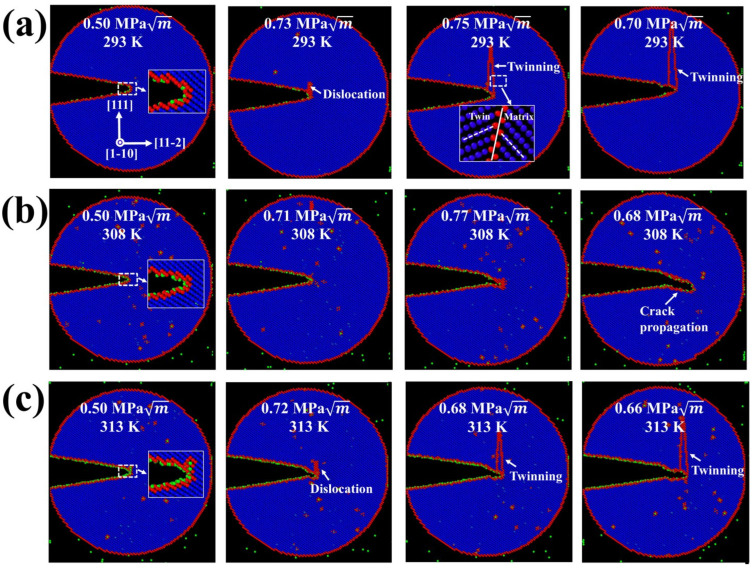
MD snapshots of crack-tip behaviour at various temperatures: images are coloured by common neighbour analysis, where BCC atoms are coloured in blue; atoms at free surfaces and defects are coloured in red; and hydrogen atoms are assigned in green: (**a**) 293 K; (**b**) 308 K; (**c**) 313 K.

Borchers et al. [35] and Wu et al. [47] suggested temperature-dependent mechanisms being associated with hydrogen-defect interactions, which might play a critical role in governing *T_HE,max_*. However, current experimental techniques cannot characterize these interactions involving nanoscale hydrogen distribution around lattice defects. Fortunately, modern simulation tools can provide nanoscale details of hydrogen interactions required to illuminate temperature effects. As expected, a mechanistic model on the basis of hydrogen accumulation around a microcrack using molecular dynamics (MD) simulations was developed and is shown in Figure 13. The model was carried out using the Large-scale Atomic/Molecular Massively Parallel Simulator (LAMMPS) (Albuquerque, NM, USA) [48]. The embedded-atom-method potential for Fe-H used in the present study was first developed by Ramasubramaniam et al. [49], and then modified by Song and Curtin [50] to eliminate the unrealistic clustering of hydrogen atoms. The specimen dimensions $L_{x}\times L_{y}\times L_{z}$ were selected as 430 Å × 480 Å × 20 Å. A crack of length 100 Å was created by removing three atomic planes along the boundary, and the periodic boundary condition was imposed along the crack front direction (*Z* axis). Given the experimentally-observed D and $C_{0}$ in Table 1, the number of hydrogen atoms around the crack tip in the model at time t can be determined by $N_{H}/L_{z}=\beta (\frac{1}{2},\frac{9}{10})\frac{2c_{0}}{a_{0}}{\left(\frac{5(1+v)D\Omega \dot{K_{I}}}{12\sqrt{2\pi }k_{B}T}t^{2}\right)}^{4/5}$ [50], where $\beta (\frac{1}{2},\frac{9}{10})$ is the beta function; the lattice constant is $a_{0}$; the partial volume of a hydrogen interstitial in iron is Ω; the Poisson’s ratio is v; the Boltzmann constant is $k_{B}$; temperature is T; and $\dot{K_{I}}$ is the loading rate. The system was initially loaded to $K_{I}=0.5\;MPa\;\sqrt{m}$ to drive hydrogen segregation around the crack tip, and was subsequently loaded by imposing successive increments of ${∆K}_{I}=0.002\;MPa\;\sqrt{m}$ every 1 × 10^−3^ ns. The Nose–Hoover method was used to keep the system temperature with a time step of 1 fs. The simulation results were illustrated by tracking the common neighbour analysis (CNA) parameter at each snapshot in Ovito [51].

**Figure 14:**
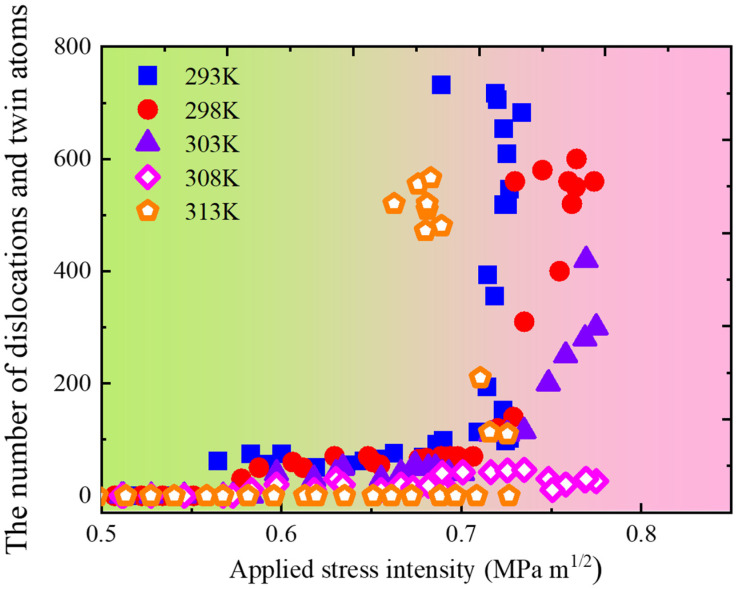
The amount of dislocations and twin atoms nearby the crack tip versus applied stress intensity.

Three observations can be made from these simulations. First, the onset of crack-tip plasticity took place by emission of dislocations and twins at 293 K. Such plasticity was likely responsible for further blunting of the crack tip, which exhibited ductile behaviour. Second, there was a brittle cleavage process at *T_HE,max_* of 308 K as the crack was seen to propagate along the slip plane without dislocation/twin emission. This behaviour could be explained in terms of increased hydrogen atoms at the crack tip as the temperature increased. The accumulated hydrogen decreased the cohesive energy of atomic planes, thereby encouraging cleavage-like failure within the framework of HEDE theory [10,11,12,47,48]. Lastly, it was observed that crack-tip behaviour was dominated by dislocation/twin emission at 313 K. This result may be surprising because the HEDE mechanism was expected to operate as hydrogen atoms at 313 K ($N_{H}/L_{z}=118\;{nm}^{-1}$) were much higher than at 308 K ($N_{H}/L_{z}=61\;{nm}^{-1}$). However, HEDE theory may fail when considering complicated plastic activity at the crack tip [52]. To unveil this, dislocation and twin atoms nearby the crack tip as a function of applied stress intensity at various temperatures were identified, traced, and counted, and are shown in Figure 14. It can be seen that at the same stress intensity, the number of dislocations and twin atoms at 313 K were much higher than those at 308 K, implying a higher local plastic activity at the crack tip. Our previous study [53] confirmed that this activity can hinder the crack from cleaving; the crack tip thus showed a ductile pattern at 313 K.

Notwithstanding the inherent shortcomings of MD simulations such as incredibly small times and length scales, our simulation model revealed the experimentally-observed ductility minimum at a temperature of 308 K, and provided an atomistic-level explanation for the hydrogen-defect interactions behind temperature-dependent HE in structural materials. Furthermore, the demonstration of *T_HE,max_* for hydrogen-induced embrittlement susceptibility using multiscale approaches (macro-scale, micro-scale, and atomistic scale) would be crucial for determining test temperatures of hydrogen-related engineering material applications.

## 4. Conclusions

The influence of hydrogen-charging current density and temperature on hydrogen permeation and HE susceptibility of 4130X steel has been studied in the present study. The central conclusions are identified as follows:

(1) With an increase in hydrogen-charging current density and temperature, the hydrogen penetration rate, hydrogen diffusion coefficient, and subsurface hydrogen concentration of 4130X steel all increase. This is mainly because the higher hydrogen-charging current density and temperature facilitate an increase in the hydrogen-charging rate, promote the cathodic reduction reaction, and result in the generation of more hydrogen atoms on the steel surface.

(2) The extent of hydrogen-induced degradation in tensile ductility, including elongation to failure and area reduction, becomes more pronounced with an increase in hydrogen-charging current density. Within the investigated temperature range of 293 K to 313 K, the HE index reaches its peak at 308 K, indicating that the critical temperature threshold for HE is 308 K.

(3) The fracture morphologies of non-charged tensile samples manifest with ductile features composed of dimples and voids. A high hydrogen-charging current density provides a higher hydrogen concentration, which facilitates the transition of fracture mode from ductile to quasi-cleavage fracture. Ductility decreases as the temperature rises to 308 K, then increases with further temperature elevation. At the critical temperature threshold of 308 K, the occurrence of quasi-cleavage planes and cracks indicates the highest HE susceptibility.

## Figures and Tables

**Table 1:** Chemical composition of domestic 4130X steel (mass fraction wt.%).

C	Mn	Si	P	S	Cr	Mo	Fe
0.280	0.860	0.270	0.007	0.003	1.000	0.230	Bal.

**Table 2:** Relationship between hydrogen permeation parameters and hydrogen-charging current density.

Charging Current Density (mA/cm^2^)	*i_∞_* (μA/cm^2^)	*D_eff_* (×10^−10^, m^2^/s)	*J_H_L* (×10^−10^, mol/m s)
10	0.59	0.49	0.61
20	0.81	0.55	0.83
30	1.38	0.56	1.42
40	1.90	0.57	1.98
50	2.63	0.59	2.71

**Table 3:** Relationship between hydrogen permeation parameters and temperature.

Temperature (K)	*i_∞_ (*μA/cm^2^)	*D_eff_* (×10^−10^, m^2^/s)	*J_H_L* (×10^−10^, mol/m s)
293	0.59	0.49	0.61
298	0.68	0.68	0.71
303	0.95	0.88	0.99
308	1.65	0.94	1.71
313	2.93	1.25	3.04

**Table 4:** Tensile test results of 4130X steel at different hydrogen-charging current densities.

Experimental Conditions	Tensile Strength (MPa)	δ (%)	Ψ (%)	$F_{H}$ (%)
Air	895.15 ± 17	10.74 ± 0.62	21.80 ± 0.68	—
10 mA/cm^2^	839.51 ± 21	6.14 ± 0.54	9.97 ± 0.63	54.28 ± 0.81
20 mA/cm^2^	682.46 ± 19	3.99 ± 0.57	6.53 ± 0.64	70.02 ± 0.79
30 mA/cm^2^	749.32 ± 14	3.62 ± 0.59	6.43 ± 0.59	70.53 ± 0.81
40 mA/cm^2^	724.16 ± 11	3.38 ± 0.42	6.07 ± 0.64	72.14 ± 0.89
50 mA/cm^2^	729.99 ± 17	3.42 ± 0.61	5.40 ± 0.69	75.22 ± 0.95

**Table 5:** Tensile test results of 4130X steel at different hydrogen-charging temperatures.

Experimental Conditions	Tensile Strength (MPa)	δ (%)	Ψ (%)	$F_{H}$ (%)
293 K, Air	895.15 ± 11	10.74 ± 0.51	21.80 ± 0.72	
293 K, Charged	839.51 ± 14	6.14 ± 0.54	9.97 ± 0.63	54.28 ± 1.84
298 K, Charged	815.17 ± 15	5.14 ± 0.55	8.31 ± 0.66	61.90 ± 1.94
303 K, Charged	763.28 ± 19	4.78 ± 0.53	8.00 ± 0.61	63.30 ± 1.71
308 K, Charged	708.31 ± 11	3.33 ± 0.54	7.22 ± 0.69	66.88 ± 1.87
313 K, Charged	731.69 ± 13	6.57 ± 0.51	10.28 ± 0.68	52.87 ± 1.76

## Data Availability

The original contributions presented in this study are included in the article. Further inquiries can be directed to the corresponding author.

## Abbreviations

The following abbreviations are used in this manuscript:

HE	Hydrogen embrittlement
HEDE	Hydrogen-enhanced decohesion
AIDE	Adsorption-induced dislocation emission
HELP	Hydrogen-enhanced localized plasticity
GB	Grain boundary
Cr-Mo	Chromium-molybdenum
SSRT	Slow strain rate tensile
